# Detection of Simulated Defect Using IR Temperature Sensors and One Point Heating

**DOI:** 10.3390/s8053345

**Published:** 2008-05-23

**Authors:** Byoung Chul Kim, Young Gun Heo, Yong Kweon Suh, Young Han Kim

**Affiliations:** 1 Department of Chemical Engineering, Dong-A University, 840 Hadan-dong, Saha-gu, Pusan, 604-714 Korea; E-mails: kbc1010@hanmail.net (B.C.K.); yhkim@mail.donga.ac.kr (Y.H.K.); 2 Department of Mechanical Engineering, Dong-A University, 840 Hadan-dong, Saha-gu, Pusan, 604-714 Korea; E-mails: ruldisgil@daum.net (Y.G.H); yksuh@dau.ac.kr (Y.K.S.)

**Keywords:** Thermography, IR thermometer, Defect Detection, Nondestructive Detection

## Abstract

Infrared temperature sensors, simple device for temperature measurement, have been modified for the measurement of temperature distribution on the metal surface in a way of nondestructive detection of defects of the object. In this study, the IR sensor system is utilized for the defect detection in a cylinder with one point heating, and the performance of the system is examined with an aluminum cylinder having a simulated defect. In addition, a 3-D conduction equation is numerically solved to compare the computed temperature profile with the measured one. The experimental outcome indicates that the defect detection is readily available with the proposed device and the point heating is practical for the applications of the defect detection. It is also found that the measured temperature distribution is comparable to the computed result from the conduction equation.

## 1. Introduction

Vessels and equipments in chemical process industries are subject to highly corrosive environment often at high temperature and high pressure requiring periodical inspection for possible defects. However, the inspection is not simple due to the opaque nature of the vessels and equipments, and a nondestructive technique has to be applied for the defect detection. Therefore, x-ray and radioactive materials have been used in the inspection in spite of serious hazardous problem of handling.

An IR thermographic device is a possible solution for the application, because it has widely been applied to various defect detections, such as the inspection of composite materials [1-3], aerospace materials [4], laminated wood [5], and thermal barrier coating [6]. The device was also used in the inspection of artificial defects in polystyrene and a plaster [7]. For the improvement of the IR thermography, a self-referencing technique for the elimination of preliminary inspection was proposed in the analysis of IR thermogram [8], and a pulsed phase thermography has been introduced [9]. Sometimes a surface coating of sensor material can replace the thermographic device [10]

When a defect is placed in a metal plate or wall of a cylindrical object, heat conduction through the object is non-uniform forming an irregular temperature distribution around the defect. The IR temperature sensor is a simple and easy device for the measurement of temperature distribution. Because its measurement range of temperature is limited and the distance between the sensor and measured object is critical to the measurement, a specially designed sensor module is necessary for the adjustment of the range and distance. The technique has been applied to the detection of simulated defect in a plate [11] and a pipe [12] showing the ability of defect detection of the system.

In this study, the IR themometer sensor is implemented to the detection of a simulated defect in a cylindrical object, same to the shape of most equipment in chemical process industries. Unlike the previous studies on the case of uniform heating to provide a temperature gradient on the surface of inspected material, one point heating is applied considering the convenience of its practical implementation. It is easy to heat a spot on a large surface of a cylindrical wall instead of uniform, linear heating for the formation of temperature distribution around the defect. A sensor module is utilized to adjust the temperature measuring range of each of five sensors used here and to maintain the distance between the sensor and object. A numerical analysis using the 3-dimensional conduction equation is also carried out to compare the measured temperature distribution with the calculated distribution.

## 2. Cylinder Model and Numerical Analysis

**Figure 1.**
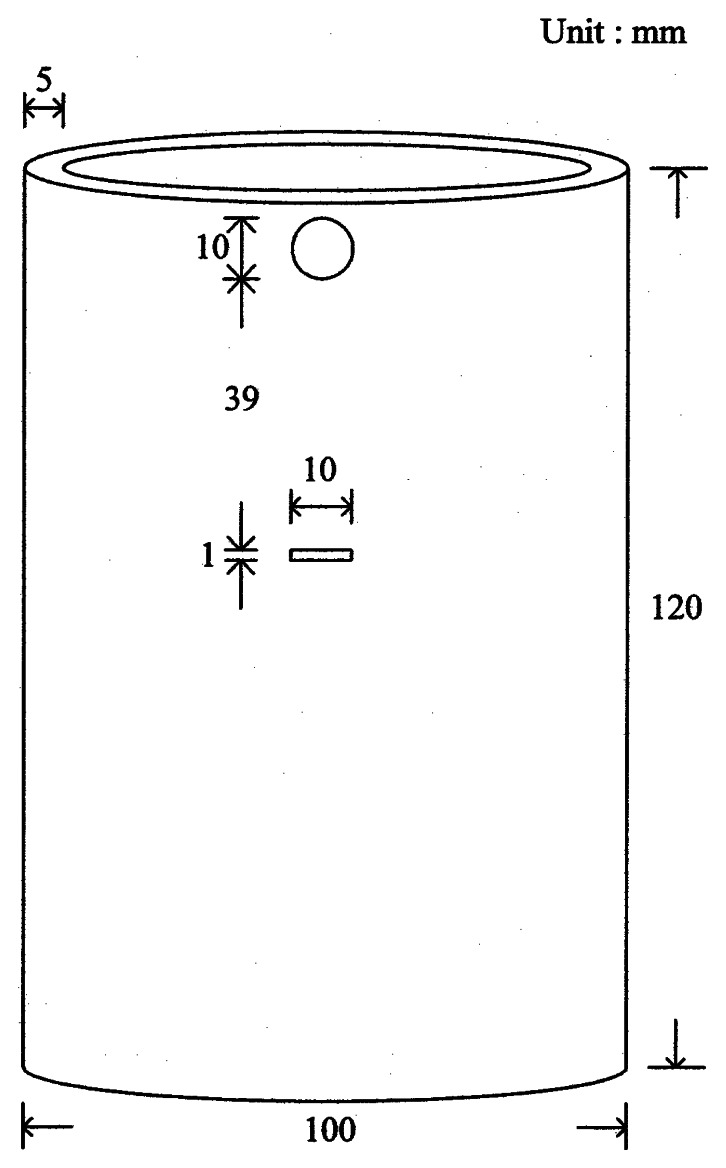
Dimension of an aluminum cylinder with a groove.

**Figure 2.**
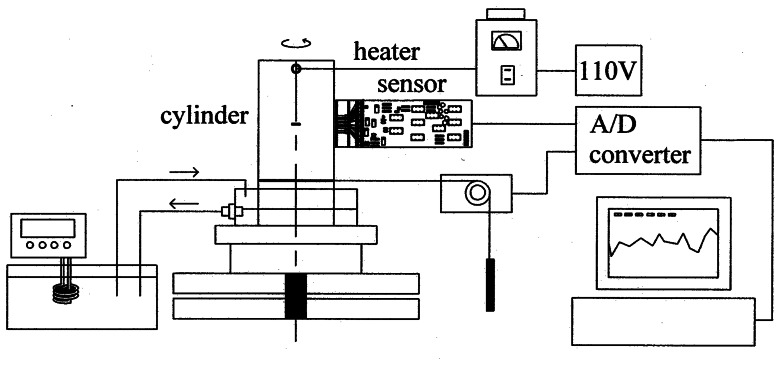
A schematic diagram of experimental setup.

An aluminum cylinder having a wall thickness of 5 mm and a diameter of 100 mm as shown in Figure 1 was utilized in the experiment, which was modeled to solve a 3-D conduction equation upon suitable boundary conditions. In the middle of the cylinder a groove of 1 mm high and 10 mm wide was drilled as a simulated defect and covered on both sides of the groove with a piece of aluminum foil to conceal the defect. During the operation of chemical processes in field the wall of a vessel is often warm and a temperature distribution is generated, in which the proposed IR thermography can be directly applied for the detection of defects without separate, external heating. However, since the temperature distribution is not provided in the sample of this study, an external heating is necessary. In order to provide the temperature distribution on the cylinder a short rod heater was inserted in the hole near the top, and a water cooler was located at the bottom as illustrated in Figure 2. In a practical application, the point heating is simple and easy to generate a temperature distribution.

The 3-D governing equation for the computation of temperature distribution for the cylinder model is

(1) $$\nabla^{2}T=0$$

**Figure 3.**
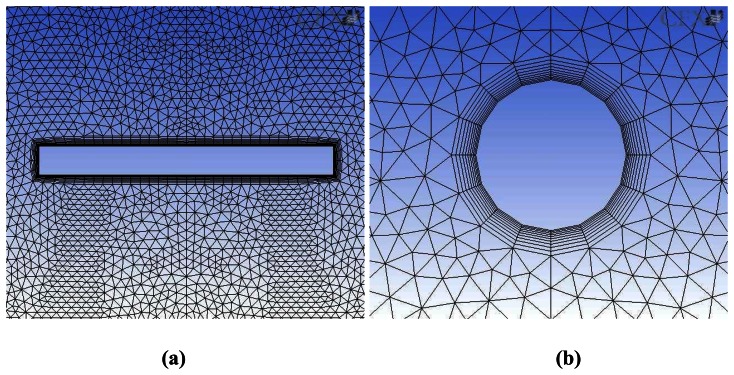
Grid structures for three-dimensional numerical simulation: **(a)** around the heat source, **(b)** around the groove.

In the computation, the heat flux at heat source is set as 8.91×10^5^ W/m^2^K, and the temperature at the bottom of the cylinder is maintained at 25 °C. All other boundaries are assumed to be adiabatic, because the heat conduction through the aluminum is much faster than the convection to the air. For the 3-D numerical solution of Eq. (1), the grid structure was made as illustrated in Figure 3. Because high temperature gradient is expected near the heat source and groove, relatively large numbers of nodes are assigned there. Total number of nodes is approximately 1,000,000. Grids are also constructed along the radial direction. Four-node grids are used around the heater, and 20-node grids around the groove. The commercial software CFX (ANSYS Inc., U.S.A., Ver. 11.0) was utilized in the numerical simulation.

## 3. Experimental

### 3.1. Preparation of sensor module

**Figure 4.**
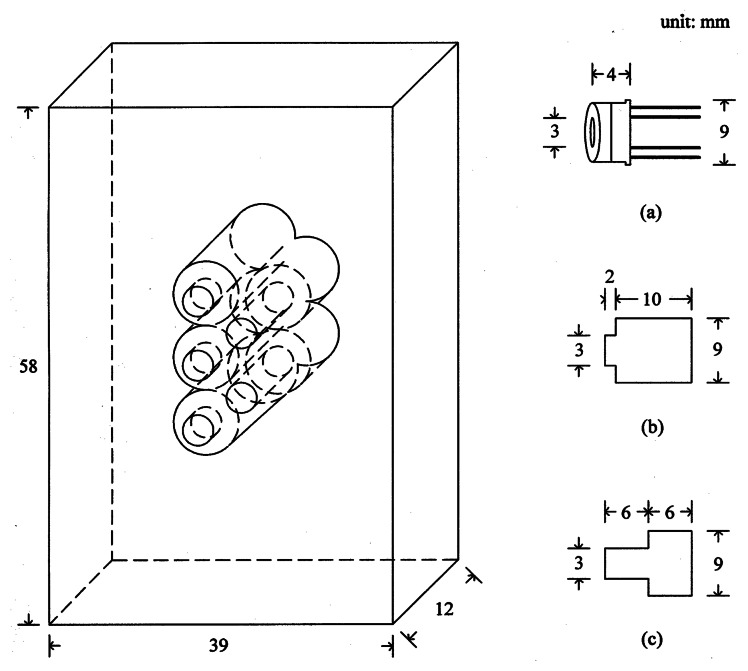
A schematic diagram of sensor holder and dimensions of sensor**(a)**, left-line hole**(b)** and right-line hole**(c)**.

In order to measure temperature distribution of the aluminum cylinder, five infrared thermometers (Heimann Sensor GmbH, Germany, Model 3129)—built in a specially designed module as described in Figure 4—were utilized in this experiment. Though the sensor has a circular optical window of 3 mm in diameter, it has 9 mm tin casing as illustrated in Figure 4a.

**Figure 5.**
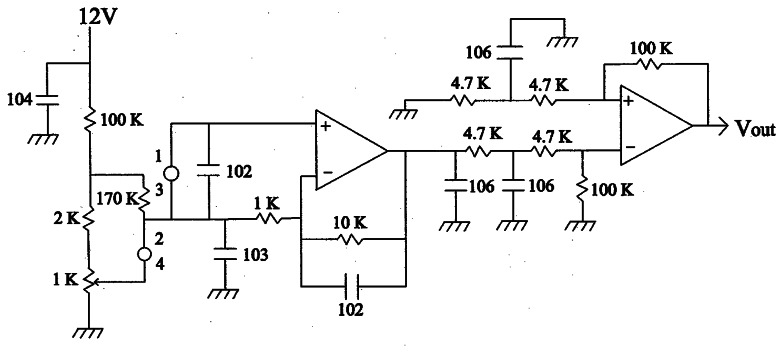
A schematic diagram of amplification circuit.

When the sensors are placed in a line, there is undetected area between two adjacent sensors due to the large casing with small optical window. For the reduction of the undetected area, a specially designed module made of bakelite was implemented as demonstrated in Figure 4. The five sensors in the module are placed in two lines. Three sensors are located in the left line with short length of viewing holes, while two sensors are in the right with long holes. Locating them in a staggered arrangement minimizes the undetected area when the module horizontally scans the cylinder surface. The sensor has an internal thermistor for temperature compensation, and a separated amplification circuit as described in Figure 5 is used for the signal processing of each sensor. To maintain a constant distance between the sensor and cylinder, the front side of the sensor module is in contact with the cylinder surface.

### 3.2. Experimental set-up

A schematic diagram of the experimental setup is shown in Figure 2. In the middle of the setup, the cylinder is placed in a cooling water pan on top of a turn table. A rod heater is installed at the top of the cylinder, and is connected to the adjusted power supply. The cooling water circulated from a thermostat (Daeil Engineering, Korea, Model DTC-312) is provided to give large temperature gradient on the cylinder. The rotational angle of the turn table is measured with a potentiometer connected to the cylinder with a string, and the measurement is transferred to a PC through an A/D converter. During the experiment, a portable IR thermometer (Raytek, U.S.A., Model Raynger IP-K) is utilized for the measurement of reference temperatures at the top and bottom of the cylinder. The IR sensors in the measurement module are calibrated with a hot aluminum plate and a thermocouple thermometer. The five voltage signals from the sensors are supplied to a PC through the A/D converter during the experiment.

### 3.3 Experimental procedure

The cylinder is adjusted to be at the center of the turn table, and it is checked that the string connected to the potentiometer for the measurement of rotational angle is properly attached to the cylinder. Then the sensor module is aligned to measure the groove temperature with the third sensor from the top. While the cooling water is supplied to the bottom of the cylinder, the heater is activated to raise the cylinder temperature. After two hours of constant supply of cooling water and heat to the cylinder, the top and bottom temperatures are periodically measured with the portable thermometer to check if the temperatures are stable. When the experiment is ready with a steady temperature distribution, the data acquisition with the PC begins and the measurement starts. While the sensor module is stationary, the turn table holding the cylinder slowly rotates at a speed of about 30 degrees per minute. The measurements of temperature and rotational angle are fed to the PC. The experimental data are stored during the measurement, and retrieved for processing after the experiment.

## 4. Results and discussion

**Figure 6.**
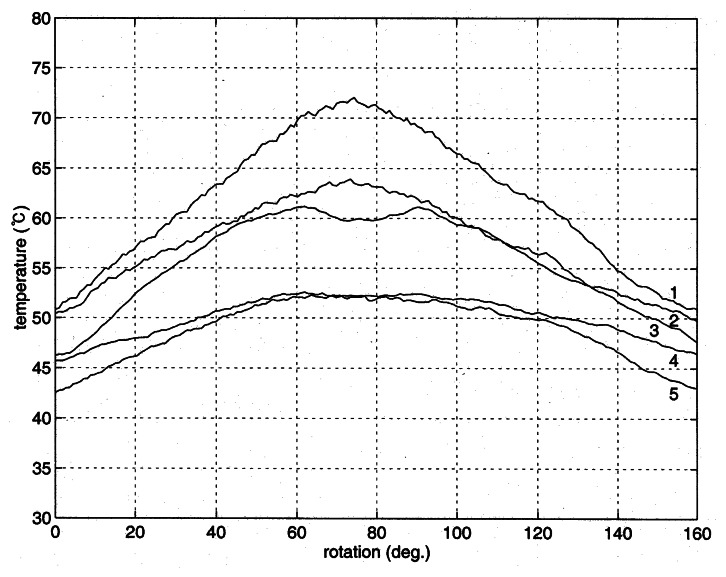
Variations of measured temperatures in the cylinder (Run-1).

When a metal object contains a defect embedded inside, the temperature distribution around the defect is distorted due to the difference of thermal conduction around the defect. The temperature on the surface of the metal object is measured here, while one point heating is provided for the generation of temperature distribution. The measurement of temperature distribution was conducted for three times. In the measurements the third sensor from the top of the sensor module was aligned to pass the center of the concealed groove. Figure 6 shows the measured temperature distribution in the experiment of run-1. The numbers on the curves are sensor position counted from the top. As expected the highest temperature was yielded at the center of the curve 1, closest to the heater, and a nearly symmetric temperature distribution was found in both sides of the cylinder. Note that the heating occurs at the center of cylinder top. A similar distribution of temperature is shown in the curve 2 except that the temperature is lower than the curve 1 due to the distance from the heater. A large drop of temperature at the center of curve 3 was detected from the defect. Because the thermal conduction through the defect does not occur, the temperature around the defect is lower than its sides clearly indicating the existence of the defect.

The lack of heat conduction from the defect also affected the temperature in the curve 4. When the curve is compared with the curves 1 and 2, the temperature in the middle is lower than expected from a no-defect cylinder. The effect of no conduction at the defect is lowered with the curve 5 as the distance from the heater increases.

**Figure 7.**
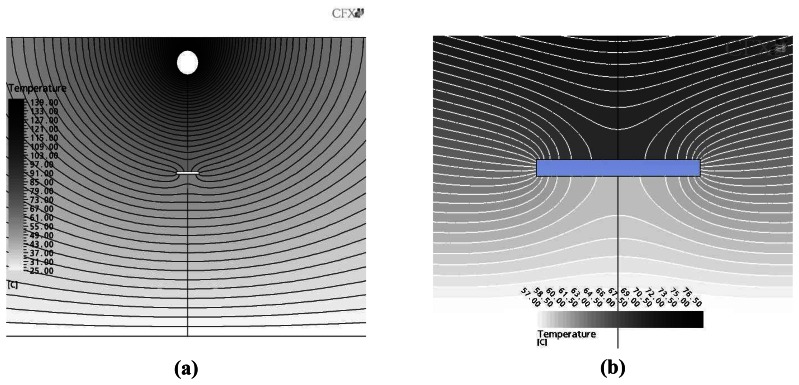
Iso-thermal contours: **(a)** global domain including the heat source and groove, **(b)** details near the groove.

The iso-thermal contour calculated from Eq. (1) is demonstrated in Figure 7. The iso-thermal temperature distribution of whole cylinder including the heater and groove is illustrated in Figure 7a, and the detailed distribution around the groove is given in Figure 7b. While large temperature gradient is observed near the heat source, the gradient around the groove is not varied much from nearby area except the disturbance caused by the groove. Therefore, the temperature measurement around the groove detects the disturbance to indicate the existence of the simulated defect.

**Figure 8.**
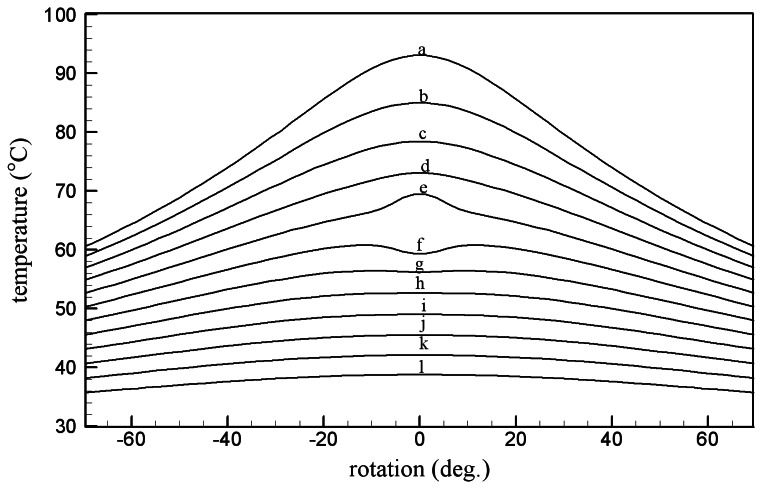
Computed temperature distribution on the cylinder surface.

**Figure 9.**
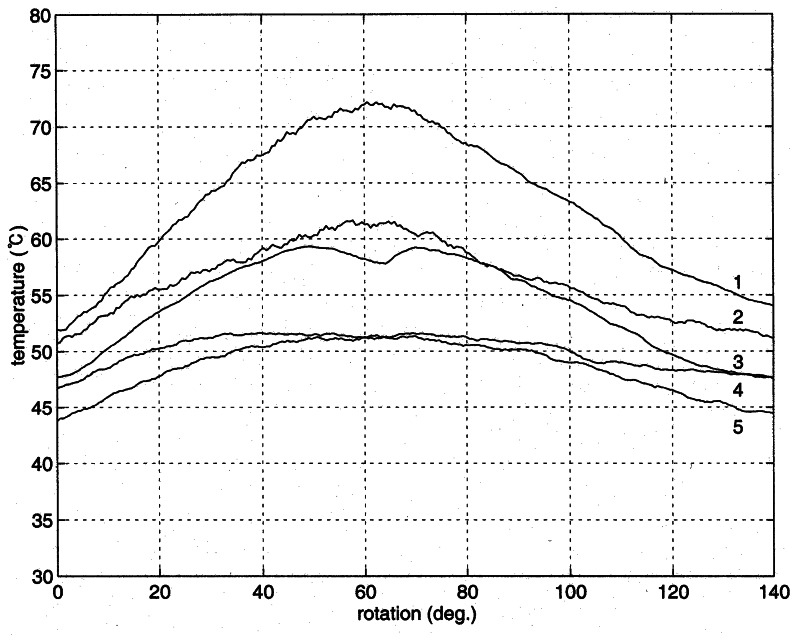
Variations of measured temperatures in the cylinder (Run-2).

**Figure 10.**
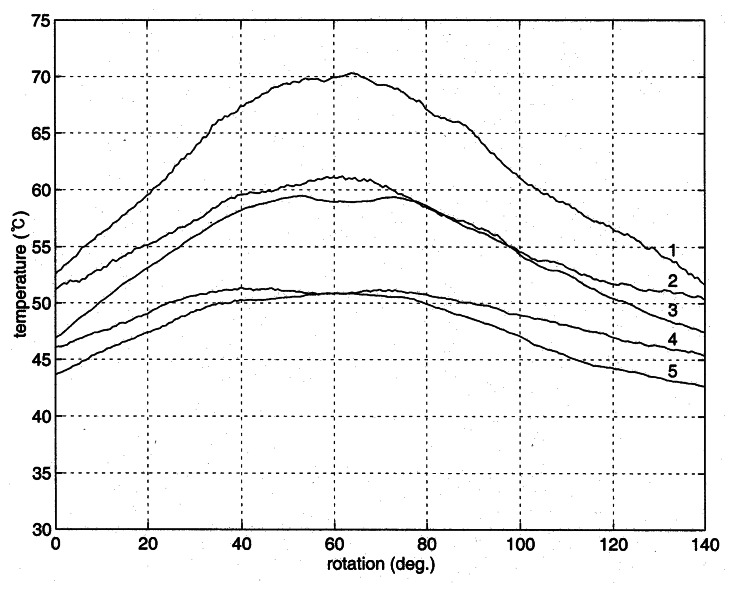
Variations of measured temperatures in the cylinder (Run-3).

For the better comparison between the measured temperature and the numerically computed distributions, the temperature around the groove is plotted in Figure 8. The patterns of temperature distribution are similar to each other. The defect is located at the center of the figure, and the temperature above the center (lines a - e) is higher than its sides as demonstrated in the curves 1 and 2 of Figure 6. However, the temperature below the defect (line f) is lower than those of the sides. When the distance from the heater increases (lines g – l), the effect of the defect diminishes as given in the curves 4 and 5 of Figure 6. Two more experiments were conducted for the inspection of reproducibility of the proposed technique, and the outcome was exhibited in Figure 9 and 10. The explanation given with Figure 6 identically applies to the results in the figures.

In the previous applications of the IR thermometers for the defect detection [11, 12] a uniform heating was utilized, but the heating is difficult to apply in a practical implementation. As demonstrated in this study, the point heating on a localized spot in a vessel wall is more practical than the uniform heating and is effective to detect a defect. In addition, the proposed procedure does not employ an x-ray or radioactive material being difficult and dangerous to handle.

## Conclusion

A set of IR thermometers was used for the detection of a simulated defect in a cylinder in order to examine if it can be used as a nondestructive defect detection device. Unlike the previous experiments, one point heating was applied because it was more practical than a uniform heating in field applications. The outcome of three experimental runs indicates that the proposed technique clearly distinguishes the defect. The measured temperature distribution was compared with the result of the numerical analysis using a 3-D conduction equation to show the similar distributions to the experimental measurements. The performance examinations exhibit that the IR defect detection system is useful in the field application of the inspection of cylindrical equipments.
